# Leveraging malaria microscopy infrastructure to diagnose common and neglected skin diseases using direct microscopy in Sumba, Indonesia

**DOI:** 10.1016/j.lanwpc.2025.101739

**Published:** 2025-11-07

**Authors:** Gladys O. Siregar, Maria Harianja, Dallas J. Smith, Messe R. Ataupah, Ruth D. Laiskodat, Claus Bøgh, Hardyanto Soebono, Marlous L. Grijsen

**Affiliations:** a Sumba Foundation, Sumba, Indonesia; b Mycotic Diseases Branch, Centers for Disease Control and Prevention, Atlanta, GA, USA; c Province Health Office, East Nusa Tenggara, Kupang, Indonesia; d Department of Dermatology and Venereology, Universitas Gadjah Mada, Yogyakarta, Indonesia; e Center for Tropical Medicine, Faculty of Medicine, Public Health and Nursing, Universitas Gadjah Mada, Yogyakarta, Indonesia; f Oxford University Clinical Research Unit Indonesia, Faculty of Medicine Universitas Indonesia, Jakarta, Indonesia; g Centre for Tropical Medicine and Global Health, Nuffield Department of Medicine, University of Oxford, UK

Common and neglected tropical skin diseases remain a substantial public health challenge and are a major global cause of disability-adjusted life years (DALYs).^1^ In low-resourced settings, access to diagnostics and specialist care are often limited, placing the responsibility of care largely on frontline healthcare workers (HCWs). Many frontline HCWs lack adequate training in identifying common and neglected skin diseases resulting in misdiagnosis and treatment delays. Addressing this gap requires innovative and context-specific approaches to mitigate the burden of skin diseases and improve the access to and quality of care.

Common and neglected skin diseases do not necessarily require advanced diagnostics, as for some conditions, like superficial fungal or bacterial infections, herpes, scabies, leprosy and chromoblastomycosis, can be made using direct microscopy. Given the limited funding available for neglected skin diseases, integration with existing health programmes is critical to achieving the 2025 World Health Assembly's Skin Diseases as a Global Public Health Priority Resolution and improving disease outcomes and prevent transmission.^2^

Sumba, a remote and underserved island in eastern Indonesia, is highly endemic for malaria and has existing microscopy infrastructure and capacity for routine diagnosis. This report highlights our experience leveraging this infrastructure to integrate diagnosis of common and neglected skin diseases, including dermatophytosis, scabies, leprosy and chromoblastomycosis.

In October 2020, we launched a teledermatology programme, in collaboration with Sumba Foundation and local authorities, supporting five community health clinics on the island. The clinics were equipped with on-site microscopes and HCWs skilled in malaria microscopy. This expertise enabled the training of HCWs in basic laboratory procedures, including microscopic examination of skin scrapings (eg. fungal infections, chromoblastomycosis, scabies) and slit-skin smears (leprosy). De-identified clinical and microscopic images of suspected individuals were submitted to an online platform using a store-and-forward approach. A team of dermatologists reviewed the cases and provided diagnostic input and treatment recommendations.^3^

As of June 2025, over 500 cases of common and neglected skin diseases identified using a combination of clinical assessments and/or direct microscopy, have been submitted to the online platform. We diagnosed 77 individuals with previously unrecognized leprosy, 10 with chromoblastomycosis, and numerous individuals with dermatophytosis.^4^^,^^5^ To ensure diagnostic accuracy, validate microscopy-based diagnoses and build capacity in local clinical and microscopic assessments, a subset of initial chromoblastomycosis cases were confirmed through histopathology.^5^ Our results provide a low-cost and feasible integration framework for the diagnosis of common and neglected skin diseases by leveraging malaria microscopy infrastructure. This framework, supported by remote teledermatology, can substantially enhance the capacity of frontline HCWs to detect common and neglected skin diseases effectively in remote settings.

Integrated approaches for diagnosing, mapping, and managing neglected tropical skin diseases (skin NTD) often focus on leveraging multiple common and neglected skin diseases. In Cameroon, Côte d’Ivoire and Ghana, an integrated active case detection and management study integrated skin NTD in yaws endemic health districts.^6^ Infrastructure and training for skin diseases in remote locations are often lacking and involve a substantial input of costs and resources. Other countries have examined the feasibility of skin screening during mass drug administration activities, which builds upon existing community infrastructure.^7^ Our integration framework builds upon a widely implemented infrastructure of national malaria control programs. Malaria has received considerable funding that has been invested in Asia, Africa, and the Americas to strengthen diagnostic capacity, with microscopy still commonly used and considered the gold standard.^8^^,^^9^ Our framework has strong potential for global applicability in malaria endemic areas, requiring minimal resources and appropriate training in direct microscopy to support the diagnosis of common and neglected skin diseases. This approach offers practical cost-effective alternatives and helps reduce reliance on more resource-intensive skin biopsies, which often pose substantial logistical challenges in low-resourced settings.

We acknowledge that the sensitivity of microscopy can vary based on several factors, including the quality of the specimen, the staining technique used, the experience of the HCW performing the procedure, and the limitations in differentiating between morphologically similar microorganisms (eg. *Mycobacterium* species). However, the diagnoses were made in the context of clinical presentation and epidemiological link.

Diagnostic integration activities require support to properly manage and follow patients during their treatment, particularly for chronic conditions like leprosy and chromoblastomycosis. Teledermatology allowed the remote support of specialist expertise to confirm diagnoses and discuss patient management and follow-up. Mobile phones and internet connectivity continue to expand to remote areas globally, other technologies such as mHealth tools or artificial intelligence could be partnered with more traditional diagnostic tools for integration activities.^10^^,^^11^

Leveraging existing malaria microscopy infrastructure can enhance the detection of certain common and neglected skin diseases. Strengthening and expanding these efforts combined with continuous collaborative efforts between government, public, and private sectors is crucial to achieve the ambitious goals set by the 2025 World Health Assembly's Skin Diseases as a Global Public Health Priority Resolution and WHO Roadmap 2021–2030 for NTD control and elimination.

## Contributors

GOS, MH, DJS and MLG drafted the manuscript. GOS, MH and CB are employed by Sumba Foundation and were involved in the clinical management and data collection of all patients. HS and MLG are dermatologists who supported the teledermatology consultations. All authors critically reviewed and approved the final version of the manuscript and provided consent for publication.

## Declaration of interests

None.
